# A retrospective study of pyogenic liver abscess focusing on *Klebsiella pneumoniae* as a primary pathogen in China from 1994 to 2015

**DOI:** 10.1038/srep38587

**Published:** 2016-12-08

**Authors:** Yun Qian, Chi Chun Wong, Sanchuan Lai, Huarong Chen, Xingkang He, Leimin Sun, Jiaguo Wu, Jiancang Zhou, Jun Yu, Weili Liu, Daoyang Zhou, Jianmin Si

**Affiliations:** 1 Department of Gastroenterology, Sir Run Run Shaw Hospital, School of Medicine, Zhejiang University, Hangzhou, China; Institute of Gastroenterology, Zhejiang University, Hangzhou, China; 2 Institute of Digestive Disease and Department of Medicine and Therapeutics, State Key Laboratory of Digestive Disease, Li Ka Shing Institute of Health Sciences, CUHK Shenzhen Research Institute, The Chinese University of Hong Kong, Hong Kong, China; 3 Department of Critical Care Medicine, Sir Run Run Shaw Hospital, School of Medicine, Zhejiang University, Hangzhou, China; 4 Emergency Department, Sir Run Run Shaw Hospital, School of Medicine, Zhejiang University, Hangzhou, China

## Abstract

Pyogenic liver abscess (PLA) is a common intra-abdominal infection in adults. In this study, we aim to explore demographic and clinical characteristics of PLA focusing on *Klebsiella pneumoniae (K. pneumoniae*) induced PLA (KP-PLA) in mainland China. A retrospective review of medical records from all patients with KP-PLA admitted to a tertiary teaching hospital over a 21-year period (1994–2015) was performed. Among 296 PLA cases with confirmed culture-positive data, *K. pneumoniae* was revealed as the predominant pathogen (*n* = 189, 63.9%), followed by *Escherichia coli (n* = 39, 13.2%). Strikingly, KP-PLA patients had a higher incidence of metabolic disorders, such as diabetes mellitus (49.7% vs. 36.4%, *P* = 0.027; odds ratio (OR): 1.725; 95% confidence interval (CI): 1.061–2.805), hypertension (38.1% vs. 19.6%, *P* = 0.001; OR: 2.520; 95% CI: 1.439–4.413), and fatty liver (32.3% vs. 14.0%, *P* = 0.001; OR: 2.923; 95% CI: 1.564–5.462) than those with non-*K. pneumoniae* induced PLA (non-KP-PLA). Moreover, patients with KP-PLA had higher susceptibility to septic metastatic infection at distant sites compared to those with non-KP-PLA (10.6% vs. 3.7%, *p* = 0.038). Our results indicate that *K. pneumoniae* is the predominant pathogen of PLA in mainland China. KP-PLA is frequently diagnosed in patients with metabolic diseases and has a higher risk for septic metastatic infection.

Pyogenic liver abscess (PLA) is a common infectious disease worldwide. A recent striking finding disclosed a much higher incidence of PLA in Taiwan (17.6 per 100,000 population) than that in the United States, Denmark and Canada (1.1–3.6 per 100,000 population)^1^,^2^,^3^,^4^. *Klebsiella pneumoniae (K. pneumoniae*) is a gram-negative bacterium. In the late 1980’s, *K. pneumoniae* induced PLA (KP-PLA) was first described in Taiwan^5^, and highlighted with subsequent case reports and clinical studies in the Asia-Pacific region^6^,^7^,^8^,^9^,^10^. Up to now, *K. pneumoniae* is considered to surpass *Escherichia coli (E. coli*) to become the predominant cause of PLA over the past three decades^11^,^12^. A definition of invasive liver abscess syndrome was proposed based on a systematic review, referring to KP-PLA with characteristic extrahepatic metastatic infection^13^. Recently, KP-PLA has been reported from North America, Europe and Oceania^14^,^15^,^16^,^17^,^18^,^19^,^20^, and was regarded as an emerging public health problem worldwide. Although KP-PLA occurs predominantly in Asian people or Asian descent, there are few reports about this disease in mainland China in spite of its large population. The aim of this study was to investigate the clinical and pathogenic characteristics of PLA in mainland China with a focus on KP-PLA by a retrospective review of 21 years’ medical records in a tertiary teaching hospital since its opening in May 1994, to better understand the clinical features of this disease and to increase awareness about KP-PLA with metastatic infections among clinicians.

## Results

### Characteristics of the study population

**Figure 1:**
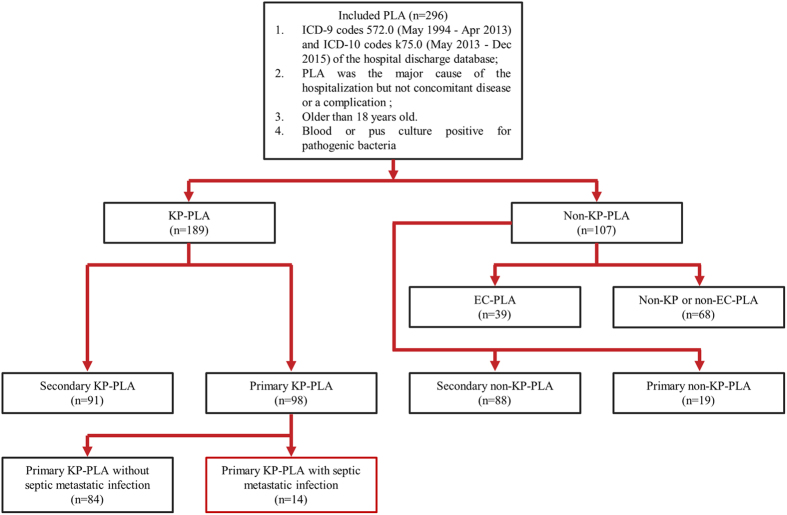
Workflow of the patients with PLA enrolled in the study during May 1994 to Dec 2015.

In total, 802 patients were identified by accessing the hospital discharge database, wherein 220 patients were excluded due to the absence of blood or pus culture results. Among the remaining 582 patients whose blood or pus was assessed by microbiology-culture, 286 were culture-negative and excluded from our analyses. Eventually, a total of 296 patients with blood or pus culture-confirmed PLA were enrolled in this single-center retrospective study (Fig. 1). Demographic characteristics and clinical features of patients with PLA are summarized in Table 1. They were male-predominant (n = 181, 61.1%) and had a mean age of 59.1 ± 12.7 years. The majority of PLA were solitary, large (size >5 cm) and localized in right hepatic lobe. Among the 296 PLA patients who had identifiable microorganisms on blood or pus culture, *K. pneumoniae* was the most commonly isolated pathogenic bacteria and was found in 63.8% (n = 189) of the culture-positive PLA patients, followed by *E. coli* (n = 39, 13.2%), *Enterococcus* (n = 20, 6.8%), *Staphylococcus* (n = 17, 5.7%), *Streptococcus* (n = 16, 5.4%), *Pseudomonas aeruginosa* (n = 15, 5.1%), Acinetobacter baumannii (n = 6, 2.0%), Corynebacterium (n = 3, 1.0%), Proteus mirabilis (n = 2, 0.7%), Klebsiella oxytoca (n = 2, 0.7%), Morganella morganii (n = 2, 0.7%), Enterobacter cloacae (n = 2, 0.7%), Citrobacter freundii complex (n = 2, 0.7%), Enterobacter aerogenes (n = 2, 0.7%), Klebsiella ozaenae (n = 1, 0.3%), Shewanella putrefaciens (n = 1, 0.3%), Pasteurella multocida (n = 1, 0.3%), Yersinia pseudotuberculosis (n = 1, 0.3%), Proteus penneri (n = 1, 0.3%) and Edwardsiella tarda (n = 1, 0.3%). Although all PLA patients were given empirical antibiotics, 256 patients (86.5%) undertook abscess drainage intervention, 36 patients (12.2%) developed septic shock, 65 patients (22.0%) were admitted to Intensive Care Unit (ICU), and 17 patients (5.7%) died eventually. As for the method of abscess drainage, 5 patients shifted to surgical drainage after failure of percutaneous drainage, and 6 patients received another CT-guided percutaneous drainage in case of dislodgement or blockage of catheters placed by previous ultrasound-guided percutaneous drainage.

There were 172 patients (58.1%) who had a medical history of hepatobiliary disease, 127 patients (42.9%) who previously had intra-abdominal trauma or surgery, and 45 patients (15.2%) who had been previously diagnosed with malignancy, especially gastrointestinal (GI) cancers, such as gastric cancer (n = 1), duodenal cancer (n = 3), colorectal cancer (n = 5), biliary tract cancer (n = 28) and pancreatic cancer (n = 4). On the whole, 179 (60.5%) cases of PLA enrolled in our study were secondary to above underlying disease, and the rest of the cases (n = 117, 39.5%) were considered as primary PLA and cryptogenic in origin.

*K. pneumoniae* isolated was susceptible to most antimicrobial agents except for ampicillin. Only 2 strains of *K. pneumoniae* showed ESBL production, which were both blood-isolated. These 2 KP-PLA patients were >70 years of age, diabetic and had a history of hypertension. During hospitalization, they developed septic shock, required ICU admission and eventually died.

### Changes in the clinical presentation of PLA during the past 21 years

In this study, the clinical features of patients with PLA, predominant pathogens causing PLA and therapeutic interventions for liver abscess drainage have changed over the past two decades (Table 2). We observed a trend towards an increased proportion of KP-PLA in all the PLA cases from 2004 to 2015 as compared to the first 10 years (64.6% vs. 58.3%), but the difference did not reach statistical significance. Furthermore, the crude annual incidences of PLA and KP-PLA were raised from 0.266% and 0.155% during the initial 10 years to 0.448% and 0.290% in next 11 years, respectively, with significant differences (*p* = 0.003 and *p* = 0.006). Although the incidence of PLA or KP-PLA had been increasing, in-hospital mortality remained stable (5.6% vs. 5.8%, *p* = 1.000), in contrast, Length of hospital stay (LOHS) reduced from 25.7 ± 19.8 days to 17.6 ± 11.3 days (*p* = 0.004). Moreover, in recent years (2004–2015) the mean size of abscess was increased (7.0 ± 2.6 cm vs. 6.0 ± 2.3 cm, p = 0.028), and more patients received percutaneous drainage (93.8% vs. 60.0%, p < 0.001), but less patients underwent surgical drainage (7.5% vs. 46.7%, p < 0.001), compared to that in 1994–2004. Compared to the initial 10 years, CT-guided percutaneous drainage (63.7% vs. 5.6%, p < 0.001) had been increasingly replaced ultrasound-guided percutaneous drainage (38.7% vs. 100.0%, p < 0.001) in the latter 10 years and was considered as the first choice intervention for PLA drainage currently.

### Comparison of patients with KP-PLA and non-KP-PLA

Given that *K. pneumoniae* was found to be the leading causative pathogen in PLA, we further explored the clinical characteristics of KP-PLA (Table 3). Comparison of patients with KP-PLA and non-KP-PLA revealed no significant differences in age or gender. KP-PLA was found to be preferentially located in the right hepatic lobe (71.7% vs. 57.9%, *p* < 0.05) and predominately cryptogenic in origin (51.9% vs. 17.8%, p < 0.001) compared with non-KP-PLA, which commonly developed secondary to underlying hepatobiliary or colorectal diseases, malignancy and intra-abdominal trauma or surgery.

Although it seemed more patients with KP-PLA were subjected to blood-culture (81.0% vs. 70.1%, *p* = 0.033), we observed a lower positive rate as compared with non-KP-PLA (33.3% vs. 56.0%, *p* < 0.001). On the contrary, KP-PLA showed a higher positive rate in liver abscess aspirate culture (97.6% vs. 90.1%, *p* = 0.020). As for treatment strategies, more KP-PLA patients underwent radiologic-guided percutaneous drainage comparing to patients with non-KP-PLA (92.8% vs. 84.4%, *p* = 0.035). Strikingly, medical chart review of KP-PLA patients revealed significant higher incidence of underlying metabolic disorders, such as diabetes mellitus (49.7% vs. 36.4%, *p* = 0.027; OR, 1.725; 95% CI, 1.061–2.805), hypertension (38.1% vs. 19.6%, *p* = 0.001; OR, 2.520; 95% CI, 1.439–4.413), and fatty liver (32.3% vs. 14.0%, *p* = 0.001; OR, 2.923; 95% CI, 1.564–5.462), as compared with patients with non-KP-PLA. In contrast, concomitant hepatobiliary disease (46.6% vs. 78.5%, *p* < 0.001; OR, 0.239; 95% CI, 0.139–0.410), malignancy (7.9% vs. 28.0%, *p* < 0.001; OR, 0.221; 95% CI, 0.113–0.435), history of hepatobiliary surgery (25.4% vs. 64.5%, *p* < 0.001; OR, 0.187; 95% CI, 0.112–0.313), and history of intra-abdominal trauma or surgery (28.6% vs. 68.2%, *p* < 0.001; OR, 0.186; CI, 0.111–0.312) were less prevalent among patients with KP-PLA. We next compared the clinical outcomes of KP-PLA and non-KP-PLA. More patients with KP-PLA developed septic shock (14.8% vs. 7.5%) and required ICU admission (24.9% vs. 16.8%). Patients with KP-PLA also tended to spend a longer time in ICU (9.0 ± 12.5 days vs. 4.0 ± 2.7 days) and hospital (18.7 ± 13.1 days vs. 18.5 ± 12.5 days). But above findings did not reach statistical significance.

Notably, patients with KP-PLA frequently developed metastatic infection at distant sites (10.6% vs. 3.7%, *p* = 0.038), leading to poor prognosis (Tables 3 and 4). Comparing the difference between metastatic KP-PLA and non-metastatic KP-PLA, we observed that metastatic KP-PLA had greater requirement for ICU support (45.0% vs. 22.5%, *p* = 0.028), longer length of ICU stay (LOIS) (11.7 ± 10.3 days vs. 8.4 ± 13.1 days, *p* = 0.040) and higher in-hospital mortality (20.0% vs, 4.1%, *p* = 0.019) (Tables 4 and 5). We also found that underlying hepatobiliary disease was less common in patients with metastatic KP-PLA as compared to non-metastatic KP-PLA (25.0% vs. 49.1%, p = 0.041) (Table 5).

### Comparison of patients with KP-PLA and *E. coli* induced PLA (EC-PLA)

There were 39 cases of EC-PLA, which accounted for 36.3% of non-KP-PLA. Patients with either KP-PLA or EC-PLA had almost the same age and a similar gender ratio. Unlike KP-PLA, of which almost half were primary and cryptogenic in origin, all EC-PLA cases were secondary to intra-abdominal trauma or surgery, and hepatobiliary diseases. As listed in Table 6, EC-PLA patients more often had a history of hepatobiliary surgery (76.9% vs. 25.4%, *p* < 0.001) and underlying hepatobiliary diseases (97.4% vs. 46.6%, *p* < 0.001) as compared with KP-PLA, implying an association between EC-PLA and concomitant hepatobiliary pathology abnormalities. Moreover, we also observed that more KP-PLA patients had underlying metabolic diseases, such as hypertension (38.1% vs. 17.9%, *p* = 0.016) and fatty liver (32.3% vs. 10.3%, *p* = 0.006) compared with EC-PLA. Overall, the prognosis appeared to be better in patients with KP-PLA than patients with EC-PLA as far as in-hospital mortality (5.9% vs. 12.8% is concerned, but this did not reach statistical significance.

### Primary KP-PLA and invasive liver abscess syndrome caused by *K. pneumoniae*

To further explore the clinical features of KP-PLA, we analyzed the subgroup primary KP-PLA and highlighted the emergence of invasive liver abscess syndrome associated with *K. pneumoniae*. As shown in Table 4, among primary KP-PLA reviewed in present study, 14 cases (14.3%) developed metastatic infection at distant sites and considered as invasive liver abscess syndrome caused by *K. pneumoniae*. Of those patients, 2 cases (14.3%) developed meningitis, 1 case (7.1%) had brain abscess, 3 cases (21.4%) grew lung abscess, 1 case (7.1%) had kidney abscess, 4 cases (28.6%) developed septic shock, 6 cases (42.9%) required ICU support, and 3 cases (21.4%) died eventually. We also noticed that 4 (4.1%) out of 98 cases of primary KP-PLA had a history of PLA, so these 4 cases were considered as recurrence of primary KP-PLA, and the range of interval of recurrence was from 7 to 96 months.

## Discussion

To the best of our knowledge, this single center retrospective study on PLA, in particular KP-PLA, covered the longest time span of all the studies reported thus far (May 1994 to Dec 2015), and provided new insights into the demographics and clinical features of patients with KP-PLA in mainland China. At our institution, 1 out of 2400 and 3700 hospital admissions were due to PLA and KP-PLA, respectively. We observed that the crude annual incidence of PLA and KP-PLA increased significantly from 1994–2004 to 2004–2015, which could be due to larger number of inpatients and improved early diagnostic techniques. In short, PLA and KP-PLA are common health problems in mainland China, which consistent with the recent epidemiological trends observed in other countries and regions^6^,^12^,^13^,^19^,^20^,^21^,^22^,^23^,^24^,^25^.

*K. pneumoniae* was considered as the main pathogen of PLA in last three decades. Most clinical studies on KP-PLA were from the Asia-Pacific region such as Taiwan, Hong Kong, Singapore and Korea^6^,^7^,^8^,^9^,^11^,^12^,^25^,^26^. To date, few study cohorts were reported from mainland China. This retrospective review of all cases of PLA at our hospital confirmed *K. pneumoniae* as the predominant pathogen of PLA followed by *E. coli*. Among KP-PLA cases, approximately half were primary PLA and cryptogenic in origin, implying that *K. pneumoniae* can invade a previously healthy liver. Whilst a report from Taiwan only discovered 1 from 160 cases of KP-PLA that was secondary to intrahepatic duct stones^11^, in our cohort a substantial number of KP-PLA cases were reported in patients with a history of intra-abdominal surgery or underlying hepatobiliary diseases. Therefore, there are considerable region differences with respect to causes that underlie development of KP-PLA. Variations in demographic composition of the study population may also contribute to different findings. Non-KP-PLA mainly occurred as secondary to intra-abdominal surgery or hepatobiliary diseases involving *E. coli*, Enterococcus, Staphylococcus, Streptococcus, *Pseudomonas aeruginosa* infection etc., consistent with previous reports^2^.

Next, we explored the patient characteristics that were associated with PLA and KP-PLA. Our data supported the views that KP-PLA mainly occurred in middle-aged men, mostly located in right hepatic lobe and had the tendency to develop distant metastasis infection^13^. More importantly, our study demonstrated that KP-PLA was significantly associated with metabolic disorders including hypertension, diabetes mellitus and fatty liver. We also showed that metastatic infection was more frequently in KP-PLA patients with hypertension, diabetes mellitus or fatty liver. Diabetes mellitus is a known predisposing factor of KP-PLA, but the incidences of diabetes mellitus in KP-PLA are different due to demographic variance, such as 15.2–25.0% in United States^15^,^16^, 27.3–39.9% in Korea^6^,^7^, 40.0% in Europe^17^,^24^, and 61.0–78.4% in Taiwan^11^,^26^,^27^. In our study, 49.7% of KP-PLA patients were diabetic. Impaired neutrophil activity and phagocytic function may contribute to relatively high frequency of KP-PLA in diabetes mellitus^11^,^26^,^28^. Up to date, limited studies observed the potential association between KP-PLA and hypertension or fatty liver, but the hidden reason still required further clarification^6^,^15^,^29^. Apart from that, quite a few PLA patients had a history of gastrointestinal cancer (41/296, 13.9%), implying a correlation between PLA and gastrointestinal cancer. Consistent with our data, Sung *et al*.^30^ showed that gastrointestinal cancer had a 4-fold higher incidence among PLA patients as compared to controls. Hence, further evaluation to rule out potential malignancies in PLA patients should be recommended in clinical practice.

Extrahepatic metastatic infection is a devastating complication for KP-PLA patients. Metastatic KP-PLA patients were in relatively severe conditions and more often admitted to ICU, but even so, about 20% patients still died eventually. To eliminate the effect of variation in demographic composition of KP-PLA study population, we traced distant metastatic infection both in cases of KP-PLA and primary KP-PLA. In this study, the incidences of extrahepatic metastatic infection in KP-PLA and primary KP-PLA were 10.6% and 14.3%, respectively, consistent with reports from other countries and regions (8–28%)^11^,^13^,^14^,^31^. A small number of primary KP-PLA cases (n = 2) developed metastatic meningitis, a rare but life-threatening complication, whose rate of death in present study was as high as 100%. Septic endophthalmitis, a major complication reported in studies from Taiwan and other regions^14^,^26^,^27^,^31^,^32^, was not observed in current study. Such discrepancy may be caused by geographic differences and inadequate follow-up of patients. In total, the in-hospital mortality in KP-PLA and primary KP-PLA with metastatic infection were 20% (4/20) and 21.4% (3/14). Given the potentially catastrophic metastatic complications associated with KP-PLA, more attention should be paid to its detection and management among clinicians.

Given the prevalence of PLA and its severe complications, particularly KP-PLA, there is a need for early detection and appropriate treatment strategy for this disease. In our study, pus culture was found to be more sensitive in detecting *K. pneumoniae* than blood culture. Thus, it is preferable to obtain pus samples from either fine needle aspiration or abscess drainage for bacteria identification prior to the use of empirical antibiotics. For those that needed abscess drainage, radiology-guided percutaneous drainage was the preferred method. As an alternative, surgical drainage was often performed in PLA patients secondary to hapatobiliary diseases or as salvage therapy in case percutaneous drainage failed. The advances in less invasive interventional radiology and endoscopic techniques^33^ will further improve the treatment of PLA. In this study, almost all of the *K. pneumoniae* isolates from patients with KP-PLA were susceptible to all the antibiotics tested except for ampicillin despite the large amount of antibiotics consumption in mainland China, which may lead to the change of gut microbiota. Considering the poor prognosis of metastatic infection complication of KP-PLA, it is of great importance for clinicians to detect the extrahepatic infection earlier and choose appropriate antibiotics with higher tissue concentration.

In present study, we observed that the blood culture positive rate (33.3%) in KP-PLA was lower than previously reported 46–61.1% by other studies^12^,^20^,^31^. We speculated that the collection time, the frequency of sampling, the amount of blood drawn for culture and number of bottles within 24 hours all might contribute to the relatively low positive rate. Furthermore, empirical antibiotics usage prior to the collection of blood culture might be another factor contributing to the low culture positive rate. We have retrospectively reviewed these patients’ medical charts and found some patients have visited local clinics or hospitals before being referred to our hospital. Thus, we cannot exclude the possibility that some of these patients were already on antibiotics prior to blood collection.

This study has several limitations. First, this study is a single center, retrospective analysis that might cause selection bias in terms of patient population admitted to this hospital and recall bias related to medical history. Second, strain specificities were not determined due to limited awareness of *K. pneumoniae* in mainland China, whilst virulence factors such as aerobactin rmpA genes, and capsular type K1 or K2 antigen have been shown to contribute to metastatic infection and severe disease^7^,^8^,^13^,^26^,^27^. Third, high percentage of monomicrobial liver abscess in Non-KP-PLA may be attributed to the presence of predominant bacteria such as *E. coli*, low positive rate of anaerobes, and may reflect inappropriate culture techniques for microorganism identification. Finally, quite a number of patients involved in present study developed septic shock and required ICU support, some predictor tools such as APACHE II score, *Glasgow* Coma Scale (GCS) and Sequential Organ Failure Assessment (SOFA) can be applied to further explore the severity and prognosis of this infectious disease.

In summary, PLA is a common infectious disease that requires hospitalization in mainland China. *K. pneumoniae* is the leading pathogen of PLA, and KP-PLA patients are at higher risk of developing metastatic complications and in-hospital mortality. Metabolic disorders, including hypertension, diabetes mellitus and fatty liver are common underlying conditions in patients with KP-PLA. Invasive liver abscess syndrome caused by *K. pneumoniae* is an emerging clinical syndrome with distant metastasis infection in mainland China and clinicians should be highly alert of its clinical characteristics to optimize patient management.

## Methods

### Study population

We performed a single-center retrospective study by compiling the inpatient medical records of all consecutive cases of PLA at the Sir Run Run Shaw Hospital, School of Medicine, Zhejiang University, Hangzhou, China, from May 1994 to December 2015. This hospital is a 2400-bed tertiary teaching hospital, which was opened in May 1994 and has an approximate annual adult admission of 46,000 during 21 years. It was the first public hospital in mainland China accredited by the Joint Commission International, a US-based, World Health Organization-authorized organization for medical quality evaluation. The list of patients with liver abscess was generated by accessing the ICD-9 (International Classification of Diseases, 9^th^ Revision) code (572.0) of the hospital discharge database from May 1994 to April 2013 and ICD-10 (International Classification of Diseases, 10^th^ Revision) code (K75.0) from May 2013 to December 2015. The initial date of discharge with a diagnosis of liver abscess was defined as the index date.

### Data collection

Patient data were retrieved from paper medical records (from May 1994 to May 2010) and electronic medical charts (from June 2010 to December 2015). The patients with PLA included in our study met the following criteria: 1) older than 18 years; 2) PLA was the major cause of the hospitalization. 3) had identifiable bacteria on blood or pus culture; We excluded patients who were 1) hospitalized due to concomitant diseases, 2) developed PLA only as a complication, 3) diagnosed with amoebic liver abscess, infected liver cyst or hepatobiliary cancer diagnosed by pathological results after discharge, and 4) previously diagnosed with PLA 30 days prior to their admission date to include only patients with newly-onset PLA.

### Ethics Statement

The institutional review board of Sir Run Run Shaw Hospital, School of Medicine, Zhejiang University approved this retrospective study protocol and waived from the need for written informed consents from these patients.

### Patient characteristics

The patients’ demographics, clinical presentations, laboratory values, radiographic findings, microbiological characteristics, treatment strategies and outcomes were collected and analyzed. LOHS, admission to ICU, LOIS (if applicable), and invasive procedures performed were documented and summarized.

### Definitions

PLA was defined as the presence of liver abscess detected by imaging technologies, together with typical clinical manifestations of infection, such as fever and right upper abdominal pain. Primary PLA was defined as PLA that occurs in the absence of a history of intra-abdominal trauma or surgery, including medical interventions such as transarterial chemoembolization (TACE) and radiofrequency ablation for treatment of hepatocellular carcinoma (HCC), underlying hepatobiliary diseases such as cholecystitis, gallstones, cholangiolithiasis, and malignant obstruction of bile ducts, underlying colorectal diseases such as polyps, adenoma and cancer, and immunosuppression due to cancer chemotherapy. On the contrary, secondary PLA was defined as PLA with above comorbidities. According to the identified pathogenic bacteria isolated from blood or liver abscess aspirate, PLA was separated into KP-PLA and non-KP-PLA, with the latter was further classified as EC-PLA and those that was neither KP-PLA nor EC-PLA. Septic metastatic infection was defined as extrahepatic infectious complications including Central Nervous System (CNS), eye, lung, muscular and skeletal system and urinary system infection, such as meningitis, brain abscess, endophthalmitis, lung abscess, necrotising fasciitis, kidney abscess and other type infection. Invasive liver abscess syndrome was defined as primary KP-PLA with septic metastatic infection at distant sites^13^.

The definition of septic shock was updated by the Sepsis Definitions Task Force in 2016 and patients with septic shock were identified with a clinical construct of sepsis with persisting hypotension requiring vasopressors to maintain MAP (mean arterial pressure) ≥65 mmHg and having a serum lactate level >2 mmol/L despite adequate fluid resuscitation^34^. Diabetes mellitus was defined as a 2 h-plasma glucose ≥200 mg/dl, a fasting plasma glucose ≥126 mg/dl or a random plasma glucose ≥200 mg/dl combined with classic symptoms of hyperglycemia^35^. Hypertension was defined as a systolic blood pressure ≥140 mmHg or a diastolic blood pressure ≥90 mmHg^36^. Fatty liver was defined as the presence of hepatic steatosis based on an ultrasound or CT scan^37^. In-hospital mortality was defined as death from any cause during hospitalization. Size of abscess was defined as the maximal cavity diameter of abscess or largest abscess when there were multiple abscesses.

### Microbiology laboratory procedures

All microbiology samples including blood and pus were processed in a central laboratory for culture. The Dade Microscan Walkaway (Dade Behring, US) (from May 1994 to Dec 2002) and the VITEK 2 system (bioMe ´rieux, Marcy l’Etoile, France) (from Jan 2003 to Dec 2015) were used to identify the bacterial isolates and perform susceptibility testing. Anti-microbial agents tested included ampicillin, ampicillin-sulbactam, amikacin, aztreonam, ciprofloxacin, cefuroxime, cefepime, cefotaxime, ceftazidime, piperacillin, piperacillin-tazobactam, cefoperazone-sulbactam, tetracycline, sulphamethoxazole-trimethoprim (SMZ-TMP) and imipenem. The extended-spectrum beta-lactamases (ESBLs) phenotype for all the collected isolates was confirmed by both a double-disk synergy test (DDST) and phenotypic confirmatory disc diffusion test (PCDDT) according to the manual of Clinical and Laboratory Standards Institute (CLSI).

### Statistical analysis

SPSS 20.0 software (SPSS Inc, Chicago, IL) was used for data analysis. Continuous variables were presented as mean ± SD and categorical data were described as frequency and percentage. Normality of the data was evaluated with Kolmogorov-Smirnov test. Independent Sample t-test was used for normally distributed data and Mann–Whitney U test was performed for non-normally distributed data. Differences were considered significant with P < 0.05. Categorical variables were compared using Pearson chi-square test or Fisher’s exact test. The presence of underlying diseases was indicated by the OR with a 95% CI. P < 0.05/n (n = comparisons number) was considered statistically significant based on the Bonferroni correction for multiple comparisons.

## Additional Information

**How to cite this article**: Qian, Y. *et al*. A retrospective study of pyogenic liver abscess focusing on *Klebsiella pneumoniae* as a primary pathogen in China from 1994 to 2015. *Sci. Rep.* **6**, 38587; doi: 10.1038/srep38587 (2016).

**Publisher’s note:** Springer Nature remains neutral with regard to jurisdictional claims in published maps and institutional affiliations.

## Figures and Tables

**Table 1:** Demographic characteristics and clinical features of patients with PLA at a tertiary teaching hospital, Zhejiang University, mainland China, from May 1994 to Dec 2015.

Variables	PLA (n = 296) N (%) or mean ± SD
Mean age (years)	59.1 ± 12.7
Gender	
Male	181 (61.1)
Female	115 (38.9)
Origin of liver abscess	
Primary liver abscess	117 (39.5)
Secondary liver abscess	179 (60.5)
Number of abscess	
Solitary liver abscess	235 (79.4)
multiple liver abscesses	61 (20.6)
Location of abscess	
Left hepatic lobe	64 (21.6)
Right hepatic lobe	199 (67.3)
≥2 lobes	33 (11.1)
Size of abscess	
Mean size (cm)	6.9 ± 2.6
Size ≤5 cm	72 (24.3)
Size >5 cm	224 (75.7)
Underlying disease	
Diabetes mellitus	133 (44.9)
Hypertension	93 (31.4)
Fatty liver	76 (25.7)
Liver cirrhosis	7 (2.4)
Hepatobiliary disease	172 (58.1)
Malignancy	45 (15.2)
History of hepatobiliary surgery	117 (39.5)
History of intra-abdominal trauma or surgery	127 (42.9)
Microbiological etiology	
KP-PLA	189 (63.8)
EC-PLA	39 (13.2)
Non-KP or EC-PLA	68 (23.0)
Method of abscess drainage	
Drainage not performed	40 (13.5)
Drainage performed	256 (86.5)
Surgical drainage	31 (12.1)
Percutaneous drainage	230 (89.8)
Ultrasound-guided drainage	100 (43.5)
CT-guided drainage	136 (59.1)
Clinical outcomes	
Septic shock	36 (12.2)
ICU admission	65 (22.0)
LOIS (days)	7.65 ± 11.0
LOHS (days)	18.6 ± 12.9
In-hospital mortality	17 (5.7)

Note: PLA, Pyogenic liver abscess; KP-PLA, *K. pneumoniae* induced PLA; EC-PLA, *E. coli* induced PLA; Non-KP or EC-PLA, non-*K. pneumoniae* or *E. coli* induced PLA; LOIS, Length of ICU stay; LOHS, Length of hospital stay.

**Table 2:** Comparison of demographic characteristics, clinical features and crude annual incidence of patients with PLA at a tertiary teaching hospital, Zhejiang University, mainland China, between 1994–2004 and 2004–2015.

Variables	May 1994-Apr 2004 PLA (n = 36) Hospital admission (n = 135.140) N (%) or mean ± SD	May 2004-Dec 2015 PLA (n = 260) Hospital admission (n = 580.210) N (%) or mean ± SD	*P* value
Mean age (years)	57.3 ± 11.4	59.4 ± 12.9	NS
Gender			NS
Male	22 (61.1)	159 (61.2)	
Female	14 (38.9)	101 (38.8)	
Origin of liver abscess			NS
Primary liver abscess	15 (41.7)	102 (39.2)	
Secondary liver abscess	21 (58.3)	158 (60.8)	
Number of abscess			NS
Solitary liver abscess	29 (80.6)	206 (79.2)	
multiple liver abscesses	7 (19.4)	54 (20.8)	
Location of abscess			NS
Left hepatic lobe	10 (27.8)	54 (20.8)	
Right hepatic lobe	22 (61.1)	177 (68.1)	
≥2 lobes	4 (11.1)	29 (11.2)	
Size of abscess			NS
Size ≤5 cm	13 (36.1)	59 (22.7)	
Size >5 cm	23 (63.9)	201 (77.3)	
Mean size (cm)	6.0 ± 2.3	7.0 ± 2.6	0.028
Underlying disease			
Diabetes mellitus	18 (50.0)	115 (44.2)	NS
Hypertension	8 (22.2)	85 (32.7)	NS
Fatty liver	5 (13.9)	71 (27.3)	NS
Hepatobiliary disease	21 (58.3)	151 (58.1)	NS
Malignancy	2 (5.6)	43 (16.5)	NS
History of hepatobiliary surgery	12 (33.3)	105 (40.4)	NS
History of intra-abdominal trauma or surgery	12 (33.3)	115 (44.2)	NS
Microbiological etiology			NS
KP-PLA	21 (58.3)	168 (64.6)	
EC-PLA	3 (8.3)	36 (13.8)	
Non-KP or EC-PLA	12 (33.3)	56 (21.5)	
Method of abscess drainage			NS
Drainage not performed	6 (16.7)	34 (13.1)	
Drainage performed	30 (83.3)	226 (86.9)	
Surgical drainage	14 (46.7)	17 (7.5)	< 0.001
Percutaneous drainage	18 (60.0)	212 (93.8)	< 0.001
Ultrasound-guided drainage	18 (100.0)	82 (38.7)	< 0.001
CT-guided drainage	1 (5.6)	135 (63.7)	< 0.001
Clinical outcomes			
Septic shock	3 (8.3)	33 (12.7)	NS
ICU admission	7 (19.4)	58 (22.3)	NS
LOIS (days)	8.0 ± 9.5	7.6 ± 11.2	NS
LOHS (days)	25.7 ± 19.8	17.6 ± 11.3	0.004
In-hospital mortality	2 (5.6)	15 (5.8)	NS
Crude annual incidence of PLA (‰)	0.266	0.448	0.003
Crude annual incidence of KP-PLA (‰)	0.155	0.290	0.006

Note: NS, not significant; PLA, Pyogenic liver abscess; KP-PLA, *K. pneumoniae* induced PLA; EC-PLA, *E. coli* induced PLA; Non-KP or EC-PLA, non-*K. pneumoniae* or E.coli induced PLA; LOIS, Length of ICU stay; LOHS, Length of hospital stay.

**Table 3:** Comparison of demographic characteristics and clinical features of patients with KP-PLA vs. non-KP-PLA at a tertiary teaching hospital, Zhejiang University, mainland China, May 1994 to December 2015.

Variables	KP-PLA (n = 189) N (%) or mean ± SD	Non-KP-PLA (n = 107) N (%) or mean ± SD	OR (95% C.I.)	*P* value
Mean age (years)	59.5 ± 13.1	58.4 ± 12.1		NS
Gender				NS
Male	115 (60.8)	66 (61.7)		
Female	74 (39.2)	41 (38.3)		
Origin of liver abscess				<0.001
Primary liver abscess	98 (51.9)	19 (17.8)		
Secondary liver abscess	91 (48.1)	88 (82.2)		
Number of abscess				NS
Solitary liver abscess	151 (79.9)	84 (78.5)		
multiple liver abscesses	38 (20.1)	23 (21.5)		
Location of abscess				0.037
Left hepatic lobe	34 (18.0)	30 (28.0)		<0.05
Right hepatic lobe	137 (71.7)	62 (57.9)		<0.05
≥2 lobes	18 (9.5)	15 (14.0)		NS
Size of abscess				0.005
Size ≤5 cm	36 (19.0)	36 (33.6)		
Size >5 cm	153 (81.0)	71 (66.4)		
Mean size (cm)	7.1 ± 2.5	6.5 ± 2.7		NS
Blood culture				
Culture performed	153 (81.0)	75 (70.1)		0.033
Positive result	51 (33.3)	42 (56.0)		<0.001
Pus culture				
Culture performed	167 (88.4)	91 (85.0)		NS
Positive result	163 (97.6)	82 (90.1)		0.020
Monomicrobial	160 (98.2)	71 (86.6)		0.001
Polymicrobial	3 (0.8)	11 (13.4)		0.001
Underlying disease				
Diabetes mellitus	94 (49.7)	39 (36.4)	1.725 (1.061–2.805)	0.027
Hypertension	72 (38.1)	21 (19.6)	2.520 (1.439–4.413)	0.001
Fatty liver	61 (32.3)	15 (14.0)	2.923 (1.564–5.462)	0.001
Hepatobiliary disease	88 (46.6)	84 (78.5)	0.239 (0.139–0.410)	<0.001
Malignancy	15 (7.9)	30 (28.0)	0.221 (0.113–0.435)	<0.001
History of hepatobiliary surgery	48 (25.4)	69 (64.5)	0.187 (0.112–0.313)	<0.001
History of intra-abdominal trauma or surgery	54 (28.6)	73 (68.2)	0.186 (0.111–0.312)	<0.001
Method of abscess drainage				NS
Drainage not performed	23 (12.2)	17 (15.9)		
Drainage performed	166 (87.8)	90 (84.1)		
Surgical drainage	16 (9.6)	15 (16.7)		NS
Percutaneous drainage	154 (92.8)	76 (84.4)		0.035
Clinical outcomes				
Septic shock	28 (14.8)	8 (7.5)		NS
ICU admission	47 (24.9)	18 (16.8)		NS
Distant metastasis	20 (10.6)	4 (3.7)		0.038
LOIS (days)	9.0 ± 12.5	4.0 ± 2.7		NS
LOHS (days)	18.7 ± 13.1	18.5 ± 12.5		NS
In-hospital mortality	11 (5.8)	6 (5.6)		NS

Note: NS, not significant; PLA, Pyogenic liver abscess; KP-PLA, *K. pneumoniae* induced PLA; Non-KP-PLA, non-*K. pneumoniae* induced PLA; Distant metastasis, extrahepatic infectious complications including Central Nervous System (CNS), eye, lung, muscular and skeletal system and urinary system infection; LOIS, Length of ICU stay; LOHS, Length of hospital stay.

**Table 4:** Characteristics of KP-PLA patients with metastatic infections from May 1994 to December 2015 (n = 20).

No.	Admission year	Age (years)	Gender	Etiology of KP-PLA	Metastatic infection site(s)	Underlying disease(s)	Complication(s)	Abscess drainage	Outcome
1	2003	42	M	primary	lung abscess	DM	none	performed	survived
2	2003	48	M	primary	^1^kidney abscess, ^2^right empyema	DM	none	performed	survived
3	2007	78	F	primary	pneumonia	fatty liver	none	performed	survived
4	2009	56	M	primary	^1^lung abscess, ^2^brain abscess	DM, fatty liver, HTN	septic shock, LOIS (8 days)	performed	survived
5	2010	36	M	primary	pneumonia	NA	None	none	survived
6	2011	55	F	primary	lung abscess	fatty liver	septic shock, LOIS (23 days)	performed	survived
7	2012	69	M	primary	^1^cellulitis of lower left leg, ^2^pneumonia	DM, HTN	none	performed	survived
8	2012	55	F	primary	meningitis	NA	septic shock, LOIS (3 days)	performed	died
9	2013	59	M	primary	bacterial prostatitis	NA	none	performed	survived
10	2013	68	F	primary	pneumonia	DM, HTN	septic shock, LOIS (32 days)	performed	died
11	2014	65	M	primary	pneumonia	DM, fatty liver	None	none	survived
12	2014	64	M	primary	right epididymitis	DM, fatty liver, HTN	LOIS (5 days)	none	survived
13	2015	66	M	primary	meningitis	DM, HTN	LOIS (8 days)	performed	died
14	2015	72	M	primary	urinary tract infection	DM	none	performed	survived
15	2005	56	M	secondary	pneumonia	DM, HTN, cholelithiasis	none	none	survived
16	2011	61	F	secondary	pneumonia	DM, HTN, cholelithiasis	septic shock, LOIS (3 days)	performed	died
17	2012	84	F	secondary	pneumonia	cholelithiasis, post-hepatobiliary surgery	LOIS (5 days)	none	survived
18	2012	70	F	secondary	pneumonia	DM, fatty liver, HTN, cholecystitis, post-intra-abdominal surgery	none	performed	survived
19	2014	76	F	secondary	pneumonia	fatty liver, HTN, cholelithiasis, post-hepatobiliary surgery	none	performed	survived
20	2014	74	F	secondary	cellulitis of lower left leg	DM, HTN, cholelithiasis	septic shock, LOIS (18 days)	performed	survived

Note: PLA, Pyogenic liver abscess; KP-PLA, *K. pneumoniae* induced PLA; DM, diabetes mellitus; HTN, hypertension; NA, not available; primary, primary PLA; secondary, secondary PLA; LOIS, Length of ICU stay.

**Table 5:** Comparison of demographic characteristics and clinical features of patients with metastatic KP-PLA vs. non-metastatic KP-PLA at a tertiary teaching hospital, Zhejiang University, mainland China, May 1994 to December 2015.

Variables	Metastatic KP-PLA (n = 20) N (%) or mean ± SD	Non-metastatic KP-PLA (n = 169) N (%) or mean ± SD	*P* value
Mean age (years)	62.7 ± 12.1	59.1 ± 13.1	NS
Gender			NS
Male	11 (55.0)	104 (61.5)	
Female	9 (45.0)	65 (38.5)	
Origin of liver abscess			NS
Primary liver abscess	14 (70.0)	84 (49.7)	
Secondary liver abscess	6 (30.0)	85 (50.3)	
Number of abscess			NS
Solitary liver abscess	17 (85.0)	134 (79.3)	
Multiple liver abscesses	3 (15.0)	35 (20.7)	
Location of abscess			NS
Left hepatic lobe	2 (10.0)	32 (18.9)	
Right hepatic lobe	17 (85.0)	120 (71.0)	
≥2 lobes	1 (5.0)	17 (10.1)	
Size of abscess			NS
Size ≤5 cm	2 (10.0)	34 (20.1)	
Size >5 cm	18 (90.0)	135 (79.9)	
Mean size (cm)	7.3 ± 2.3	7.0 ± 2.5	NS
Underlying disease			
Diabetes mellitus	13 (65.0)	81 (47.9)	NS
Hypertension	10 (50.0)	62 (36.7)	NS
Fatty liver	7 (35.0)	54 (32.0)	NS
Hepatobiliary disease	5 (25.0)	83 (49.1)	0.041
Malignancy	0 (0.0)	15 (8.9)	NS
History of hepatobiliary surgery	2 (10.0)	46 (27.2)	NS
History of intra-abdominal trauma or surgery	3 (15.0)	51 (30.2)	NS
Method of abscess drainage			NS
Drainage not performed	5 (25.0)	18 (10.7)	
Drainage performed	15 (75.0)	151 (89.3)	
Clinical outcomes			
Septic shock	6 (30.0)	22 (13.0)	NS
ICU admission	9 (45.0)	38 (22.5)	0.028
LOIS (days)	11.7 ± 10.3	8.4 ± 13.1	0.040
LOHS (days)	20.7 ± 13.7	18.5 ± 13.1	NS
In-hospital mortality	4 (20.0)	7 (4.1)	0.019

Note: NS, not significant; PLA, Pyogenic liver abscess; KP-PLA, *K. pneumoniae* induced PLA; Metastatic KP-PLA, KP-PLA with extrahepatic infectious complications; Non-metastatic KP-PLA, KP-PLA without extrahepatic infectious complications; LOIS, Length of ICU stay; LOHS, Length of hospital stay.

**Table 6:** Comparison of demographic characteristics and clinical features of patients with KP-PLA vs. EC-PLA at a tertiary teaching hospital, Zhejiang University, mainland China, May 1994 to December 2015.

Variables	KP-PLA (n = 189) N (%) or mean ± SD	EC-PLA (n = 39) N (%) or mean ± SD	*P* value
Mean age (years)	59.5 ± 13.1	59.7 ± 12.2	NS
Gender			NS
Male	115 (60.8)	20 (51.3)	
Female	74 (39.2)	19 (48.7)	
Origin of liver abscess			<0.001
Primary liver abscess	98 (51.9)	0 (0.0)	
Secondary liver abscess	91 (48.1)	100 (100.0)	
Number of abscess			NS
Solitary liver abscess	151 (79.9)	34 (87.2)	
Multiple liver abscesses	38 (20.1)	5 (12.8)	
Location of abscess			NS
Left hepatic lobe	34 (18.0)	13 (33.3)	
Right hepatic lobe	137 (72.5)	23 (59.0)	
≥2 lobes	18 (9.5)	3 (7.7)	
Size of abscess			NS
Size ≤5 cm	36 (19.0)	12 (30.8)	
Size >5 cm	153 (81.0)	27 (69.2)	
Mean size (cm)	7.1 ± 2.5	6.8 ± 2.7	NS
Blood culture			
Culture performed	153 (81.0)	27 (69.2)	NS
Positive result	51 (33.3)	16 (59.3)	0.010
Pus culture			
Culture performed	167 (88.4)	33 (84.6)	NS
Positive result	163 (97.6)	30 (90.9)	NS
Monomicrobial	160 (98.2)	25 (83.3)	0.003
Polymicrobial	3 (1.8)	5 (16.7)	0.003
Underlying disease			
Diabetes mellitus	94 (49.7)	13 (33.3)	NS
Hypertension	72 (38.1)	7 (17.9)	0.016
Fatty liver	61 (32.3)	4 (10.3)	0.006
Hepatobiliary disease	88 (46.6)	38 (97.4)	<0.001
Malignancy	15 (7.9)	13 (33.3)	<0.001
History of hepatobiliary surgery	48 (25.4)	30 (76.9)	<0.001
History of intra-abdominal trauma or surgery	54 (28.6)	31 (79.5)	<0.001
Method of abscess drainage			NS
Drainage not performed	23 (12.2)	4 (10.3)	
Drainage performed	166 (87.8))	35 (89.7)	
Clinical outcomes			
Septic shock	28 (14.8)	4 (10.3)	NS
ICU admission	47 (24.9)	10 (25.6)	NS
Distant metastasis	20 (10.6)	1 (2.6)	NS
LOIS (days)	9.0 ± 12.5	3.7 ± 2.6	NS
LOHS (days)	18.7 ± 13.1	16.9 ± 9.1	NS
In-hospital mortality	11 (5.8)	5 (12.8)	NS

Note: NS, not significant; PLA, Pyogenic liver abscess; KP-PLA, *K. pneumoniae* induced PLA; EC-PLA, *E. coli* induced PLA; Distant metastasis, extrahepatic infectious complications including Central Nervous System (CNS), eye, lung, muscular and skeletal system and urinary system infection; LOIS, Length of ICU stay; LOHS, Length of hospital stay.
